# What makes the kidney so tolerant?

**DOI:** 10.1172/JCI183501

**Published:** 2024-08-15

**Authors:** Paolo Molinari, Paolo Cravedi

**Affiliations:** 1 Translational Transplant Research Center (TTRC), Icahn School of Medicine at Mount Sinai, New York, New York, USA.; 2 Unit of Nephrology, Dialysis and Kidney Transplantation, Fondazione IRCCS Ca’ Granda Ospedale Maggiore Policlinico di Milano, Milan, Italy.

## Abstract

Various organ allografts differ in their propensity to be spontaneously accepted without any immunosuppressive treatment. Understanding the mechanisms behind these differences can aid in managing alloimmune responses in general. C57BL/6 mice naturally accept DBA/2J kidney allografts, forming tertiary lymphoid organs containing regulatory T cells (rTLOs), crucial for graft acceptance. In this issue of the *JCI*, Yokose and colleagues revealed that rTLOs promote conversion of cytotoxic alloreactive CD8^+^ T cells into exhausted/regulatory ones, through an IFN-γ–mediated mechanism. Their study provides insights into tolerance development that could help promote the acceptance of grafts at higher risk of rejection.

## Not all transplanted organs are created equal

Kidney and liver allografts are capable of actively contributing to the induction and maintenance of immunological tolerance (1, 2), while heart and lung allografts are generally considered “tolerance-resistant” (3). Understanding the mechanisms behind these organ-specific differences could help develop strategies to monitor and tailor alloimmune responses against transplanted organs in general.

C57BL/6 (B6) mice naturally accept allogeneic DBA/2J kidneys and form intragraft lymphocytic aggregates around the small vessels of the graft, resembling the tertiary lymphoid organs (TLOs) found in solid tumors (4, 5). These structures, containing Foxp3^+^ regulatory T cells (Tregs), are consistently found in accepted kidney allografts and are absent in rejected ones (5). Consistently, accepted grafts with regulatory TLOs (rTLOs) show, together with increased transcripts of Treg-associated genes (4, 5), markers of T cell exhaustion (PD-1, LAG3, and TIM3) (4). Until now, the mechanisms responsible for rTLO formation and how they promote acceptance of kidney allografts were unclear.

## Why are kidney grafts spontaneously accepted in mice?

In this issue of the *JCI*, Yokose et al. (6) unravel important mechanisms responsible for spontaneous acceptance of kidney allografts in mice. They performed serial single-cell RNA-seq (scRNA-seq) analyses of immune infiltrates isolated from spontaneously accepted kidneys. During the first week after transplantation, infiltrating CD8^+^ T cells expressed genes related to cytotoxicity (including *Gzmb* and *Ifng*) and proliferation (including *Top2a* and *Ki67*), while from the third week these cells started expressing more genes related to regulation (including *Fgl2* and *Il2rb*) and exhaustion (including *Pdcd1* and *Lag3*). Importantly, CD122(*Il2r*)^+^CD8^+^ T cells isolated from tolerated kidney grafts are able to inhibit naive CD4^+^ T cell proliferation in vitro, defining them as de facto CD8^+^ Tregs. Previous studies have shown that Foxp3^+^ Tregs in rTLOs are critical in downregulating alloimmune response (4, 5, 7), but the role of CD8^+^ Tregs in kidney acceptance was unclear. When transplanted into CD8-KO recipients, DBA/2J kidneys were still accepted without immunosuppression, suggesting that CD122(*Il2r*)^+^CD8^+^ Tregs are not necessary for acceptance of the transplanted kidney (6). However, CD8-KO mice may have impaired alloreactive responses overall (8). Selective depletion or adoptive transfer experiments of CD122(*Il2r*)^+^CD8^+^ Tregs are needed to establish the role of these cells in spontaneously acceptance of kidney grafts and, possibly, in prolonging survival of grafts that are normally rejected.

**Figure 1:**
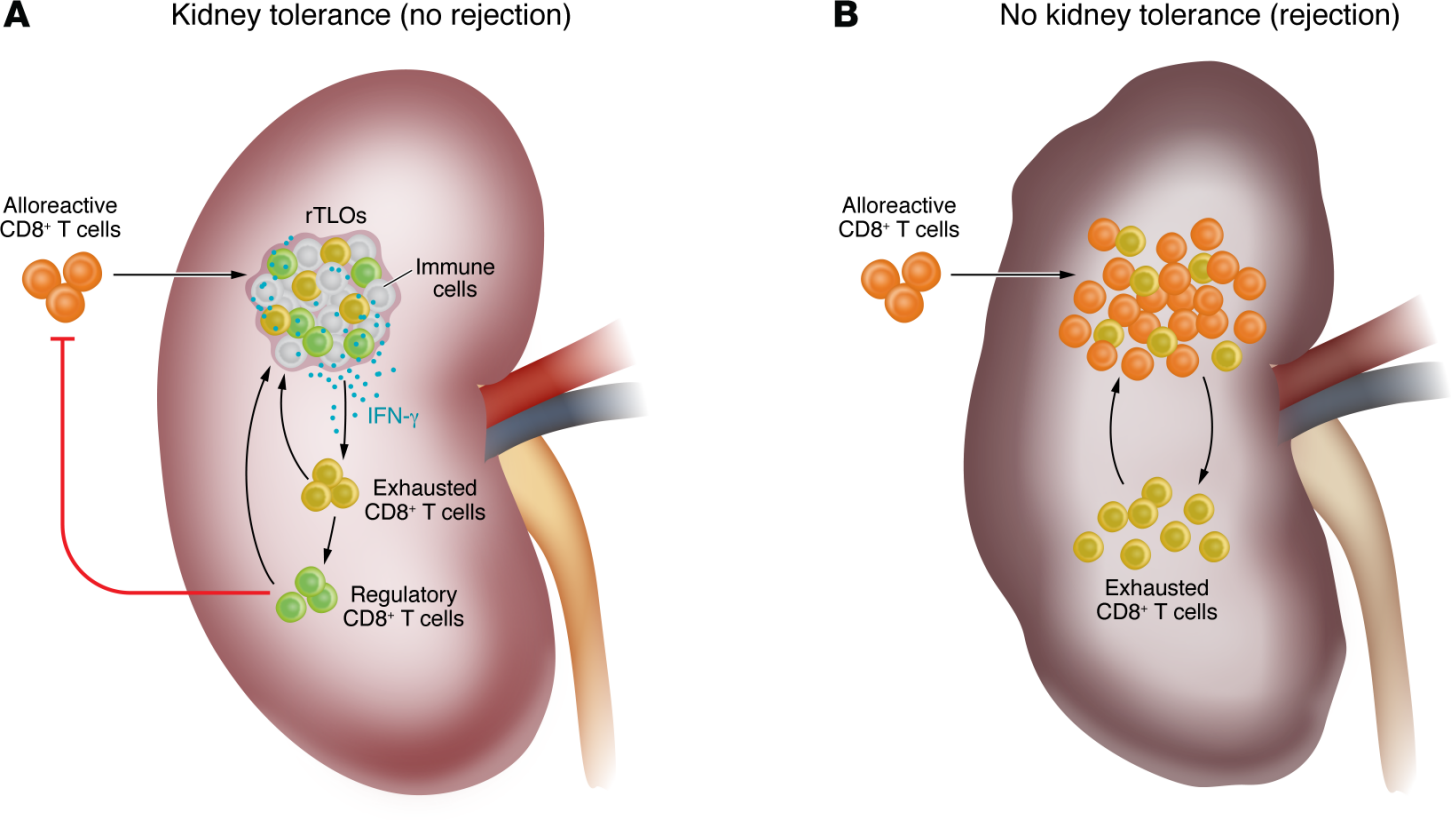
Regulatory T cell–rich tertiary lymphoid organs have a role in kidney transplant tolerance and rejection. (**A**) Regulatory T cell–rich tertiary lymphoid organs (rTLOs) form in the context of a kidney transplant that is tolerated by the recipient. These structures contain various types of T lymphocytes that, through the release of IFN-γ, promote the conversion of alloreactive CD8^+^ T cells into exhausted and then regulatory cells. (**B**) In contrast, kidneys that undergo rejection do not form rTLOs, but instead show diffuse and unorganized lymphocytic infiltrates, primarily composed of alloreactive CD8^+^ T cells, and to a lesser extent, exhausted CD8^+^ T cells.

Yokose and authors next used pseudotime analyses to show that the transcriptional profile of CD8^+^ T cells infiltrating the accepted kidneys evolved through a series of stages, from cytotoxic to exhausted, and then to regulatory, while in the rejected kidneys they transitioned from cytotoxic to exhausted without becoming regulatory. Of note, when DBA/2J kidneys were transplanted into mice lacking the PD-1 exhaustion marker and checkpoint molecule (PD-1–KO), the organ was rapidly rejected without rTLO formation, supporting the importance of alloreactive T cell exhaustion for kidney allograft acceptance. This finding is consistent with data in mice (9) and humans (10) showing that prolonged survival of transplanted grafts is associated with increased T cell exhaustion (Figure 1).

Intriguingly, when alloreactive CD8^+^CD45.1^+^ T lymphocytes were adoptively transferred after the development of tolerance, the survival of the transplanted organ was not compromised, and transferred CD8^+^ T lymphocytes showed a clear transition to CD122(*Il2r*)^+^CD8^+^ Tregs two weeks after transfer. In contrast, when alloreactive CD8^+^CD45.1^+^ lymphocytes were transferred before transplantation, they led to rejection and prevented rTLO development, indicating that intragraft rTLOs are needed for reprogramming effector T cells. Of note, alloreactive T cell reprogramming seems to happen within the kidney graft, not in secondary lymphatic organs, as suggested by the fact that CD8^+^ cells in the spleen showed milder transcriptional changes than the ones obtained from the kidney graft. This is important information to be considered when interrogating peripheral lymphocytes to obtain information about immunological events occurring during graft acceptance.

Finally, Yokose and authors investigated the key mechanism responsible for CD8^+^ Treg induction in the kidney graft. scRNA-seq data showed that *Ifng* transcription was increased in T lymphocytes infiltrating the accepted kidney (mainly in rTLOs). Lymphocytes isolated from accepted kidneys showed increased production of IFN-γ, even without stimulation. When DBA/2J kidneys were transplanted into B6.*Ifng^–/–^* (IFN-γ–KO) mice, grafts were rejected. Similarly, adoptive transfer of alloreactive T cells lacking the IFN-γ receptor (IFNGR-KO) was associated with histological signs of rejection. Altogether, these data indicate that intrarenal IFN-γ production plays a critical role in promoting the formation of CD122(*Il2r*)^+^CD8^+^ Tregs and kidney graft acceptance (6) (Figure 1).

## Impact on transplant medicine and future directions

In humans, kidney grafts are not spontaneously tolerated. However, there are rare individuals who successfully withdraw immunosuppression without rejecting their grafts. Long-term graft acceptance in the absence of ongoing immunosuppression has been often associated with B cell and Treg signatures, but the mechanisms behind “operational tolerance” have not been fully characterized (11, 12).

The present work indicates that kidney graft acceptance requires an initial phase of T cell activation and the production of IFN-γ — presumably by Tregs in rTLOs — is critical for the induction of T cell exhaustion and for the emergence of their regulatory function (6). Intriguingly, *Ifng* deletion accelerates rejection of otherwise spontaneously accepted liver allografts in mice (13) and *Ifng^–/–^* animals are resistant to the induction of tolerance to skin and heart allografts through costimulation blockade (14). Therefore, while being a key proinflammatory cytokine (15), IFN-γ plays a critical role in tolerance induction and maintenance. Does this apply to humans as well? Possibly yes, but it needs to be tested further. Acute rejection, a condition where IFN-γ production is high, is a strong stimulus for Treg induction within the graft (16, 17). Organs undergoing rejection have a marked increase in the percentage of Tregs in the infiltrate that can be also detected in urine from patients (17). The work by Yokose et al. (6) sets the stage for future studies aimed at understanding the molecular mechanisms responsible for IFN-γ–driven conversion of alloreactive T cells into ones with exhausted/regulatory function.

Current histological interpretation of graft biopsies is largely based on the assumption that infiltrating leukocytes exert a detrimental effect on graft parenchymal cells (18). Yokose et al. (6) clearly show that intragraft immune cells can exert also regulatory or protective effects. Phenotypic and transcriptional characterization of graft infiltrates in organ transplant recipients is an area of research that will likely allow us to differentiate between infiltrates with accepting versus rejecting signatures (19). This information will be particularly important for graft biopsies performed in organ transplant recipients with stable function, where the lack of signs of graft injury makes the interpretation of immune infiltrates challenging.

Another key message of Yokose et al. (6) relates to the persistence of alloreactive cells that remain in the accepted graft in an exhausted/regulatory form. The stability of the regulatory program of these cells has not been fully characterized. Although transfer of alloreactive T cells does not lead to rejection, it is possible that, in the presence of proinflammatory signals (e.g., infections), these cells convert into effector ones. This is an important possibility that merits further investigation.

Intriguingly, while heart grafts are promptly rejected, combined heart and kidney transplants are accepted long-term in mice (20). This suggests the existence of circulating factor(s) that can extend acceptance of kidneys to other cotransplanted, more immunogenic, grafts. The kidney is the source of various molecules with pro-tolerogenic function, including, among others, active vitamin D and erythropoietin (21, 22). More recently, data have been generated showing that erythropoietin, produced by peritubular fibroblasts in response to hypoxia, favors Treg induction and promotes long-term kidney and heart graft acceptance (23). Whether renal production of these and other molecules participates into the pro-tolerogenic effects of the kidney and whether administration of such molecules extends survival of other organ transplants has not been fully established in humans.

## Implications for cancer immunology

Yokose et al. (6) focused their last series of experiments to demonstrate that the same tolerance mechanisms that occur in transplanted kidneys also apply to tumors. TLOs were detectable in models of pancreatic and colorectal cancer and were similar to rTLOs. The lymphoid organs were rich in Foxp3^+^CD4^+^ and CD122(*Il2r*)^+^CD8^+^ Tregs, and showed increased *Ifng* expression. Also, within the tumor microenvironment, the formation of TLOs drove the differentiation of CD4^+^ and CD8^+^ naive T cells toward a regulatory phenotype.

This finding is a further demonstration that transplant and tumor immunology have numerous features in common (24). Even if the clinical goals are opposite (graft acceptance versus tumor rejection), understanding the mechanisms that regulate interaction between immune and parenchymal cells in these two settings is likely to help both transplant and tumor research. This paper is a new step in the right direction.

## Version 1 08/15/2024

Electronic publication
